# Gastrinoma: A Case of Chronic Diarrhea

**DOI:** 10.7759/cureus.81253

**Published:** 2025-03-26

**Authors:** Putha Venkata Lakshmi Sahithi, Venugopal Prudvesh Kumar Reddy Nandi, Yashwanth Kandagaddala, Vinusha Mulagapaka, Lourdhu Pragna Onteddu, Nirmal K Onteddu

**Affiliations:** 1 Internal Medicine, P.E.S. Institute of Medical Sciences and Research, Kuppam, IND; 2 Radiology, Tata Memorial Centre, Mumbai, IND; 3 Internal Medicine, Jagadguru Jayadeva Murugarajendra Medical College, Davanagere, IND; 4 Health Sciences, University of Ottawa, Ottawa, CAN; 5 Internal Medicine, University of Florida College of Medicine, Jacksonville, USA

**Keywords:** diarrhea, gastrinoma, neuroendocrine tumor, zes, zollinger-ellison syndrome

## Abstract

Gastrinoma is a rare neuroendocrine tumor characterized by the hypersecretion of gastrin. Early diagnosis is challenging due to its rarity, and patients present to the outpatient department with nonspecific complaints such as diarrhea, weight loss, and fatigue, which often lead to a broad differential diagnosis. This report presents the case of a 59-year-old female who has come to the internal medicine department for a workup of chronic diarrhea, low-grade fever, 8kg weight loss, and severe reflux of eight months duration, not responding to empiric therapy. After multiple hospital visits and evaluations, she was found to have duodenal gastrinoma. This case report aims to create awareness and underscores the importance of considering neuroendocrine tumors as an initial differential diagnosis for patients with nonspecific or unexplained gastrointestinal symptoms to facilitate early diagnosis.

## Introduction

Gastrinomas are neuroendocrine tumors that are characterized by hypersecretion of gastrin, resulting in Zollinger Ellison syndrome (ZES), of which nearly 80% are sporadic and the other 20-25% are associated with multiple endocrine neoplasia syndrome (MEN) type 1 [1,2]. The incidence of gastrinoma is approximately 1-3 cases per year for every 1 million individuals [3]. Approximately 50-60% of gastrinoma cases are malignant. ZES is rare and frequently diagnosed in the 5th decade of life, with a higher incidence in males [4]. The classic triad of ZES includes high fasting serum gastrin levels, hypersecretion of gastric acid, and associated peptic ulcer disease and diarrhea [5]. Although the majority of cases present with peptic ulcer disease manifesting as abdominal pain and diarrhea, around 10% of the patients may present with diarrhea as the sole symptom [6]. Gastrointestinal bleeding can also be the initial presentation in one-fourth of patients. Renal stones are more commonly seen in patients with gastrinoma associated with MEN Type 1. Many ZES patients remain undiagnosed due to their symptoms mimicking ordinary peptic ulcer disease [7]. Diagnosis is based on characteristic laboratory and histological findings. Fasting serum gastrin level is a valuable screening test in patients with unexplained chronic diarrhea to evaluate for gastrinoma. It is shown to have a sensitivity of >99% and reduces diagnostic delays [7]. In a typical patient, the fasting gastrin levels are less than 100 pg/ml; however, a suspicion for gastrinoma is higher if the fasting gastrin levels are greater than 300 pg/ml. Gastrin levels greater than 1000 pg/ml with a gastric pH of less than 2 are considered diagnostic of gastrinoma [5,8]. The 5-year survival rate of gastrinoma is 20-40% in the metastatic cases [3].

## Case presentation

We report a case of a 59-year-old female without comorbidities presented for painless diarrhea of one week duration with low-grade fever, and episodic epigastric pain. The patient denied smoking, alcohol, or drug abuse. There was no significant family history. On presentation, the patient was conscious, coherent, and cooperative. The blood pressure was 110/80mmHg, heart rate was 101 beats per minute, respiratory rate was 20 cycles per minute, and body temperature was 37 degrees Celsius. Eastern Cooperative Oncology Group's performance status was zero. On physical examination, there was no abdominal tenderness or palpable abdominal mass. Heart and lungs were normal to auscultation. Basic investigations, including but not limited to complete blood count, basic metabolic panel, liver panel, and stool analysis, were unremarkable. The patient’s symptoms were attributed to be related to infectious diarrhea, and she was managed conservatively with anti-diarrheal agents and proton pump inhibitors (PPI).

**Figure 1:**
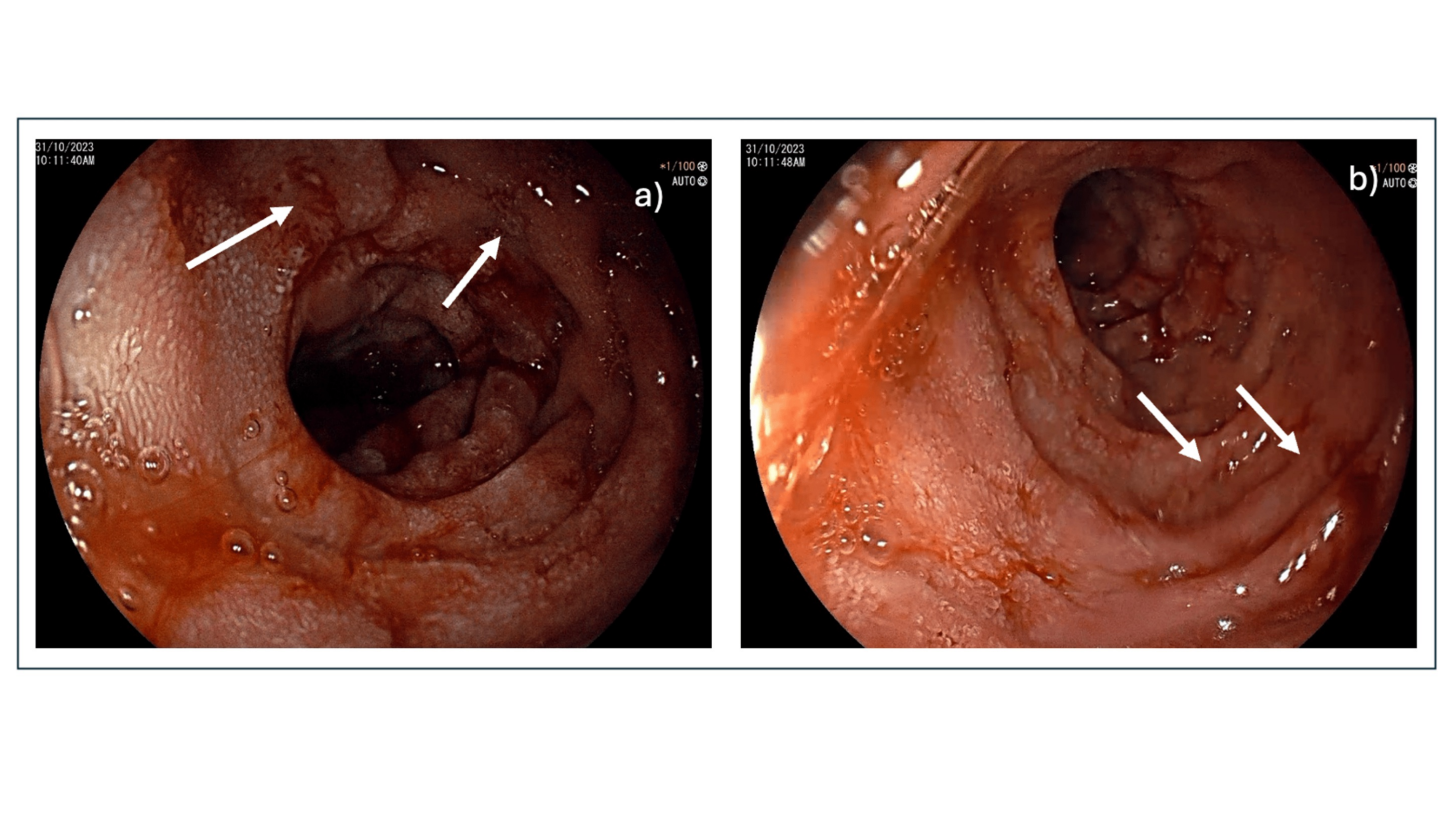
(a) Upper gastrointestinal endoscopy showing prominent mucosal folds (arrows) (b) Upper gastrointestinal endoscopy showing multiple ulcers within duodenum (arrows)

However, the patient presented again after two months with persistent symptoms, which prompted an upper gastrointestinal (UGI) endoscopy. It showed esophagitis and distal gastric hyperemia with prominent mucosal folds (Figure 1a). A rapid urease test was positive, indicating *Helicobacter pylori (H. pylori)* gastritis, and she was treated with proton pump inhibitors and antibiotics.

The patient was admitted after a few months due to symptom progression associated with anorexia and weight loss of 8 kilograms. The patient was worked up again in detail. The complete blood count, basic metabolic panel, and liver panel were within normal limits and relatively unchanged from the initial presentation. The stool osmotic gap was within normal limits, and the stool sample was negative for *Clostridium difficile* toxin, blood, and leukocytes. Fecal fat analysis showed normal fat quantity. A CT scan of the abdomen was unremarkable. UGI endoscopy demonstrated esophagitis with multiple ulcers over the folds in the duodenum and jejunum and a normal colonoscopy (Figure 1b).

**Figure 2:**
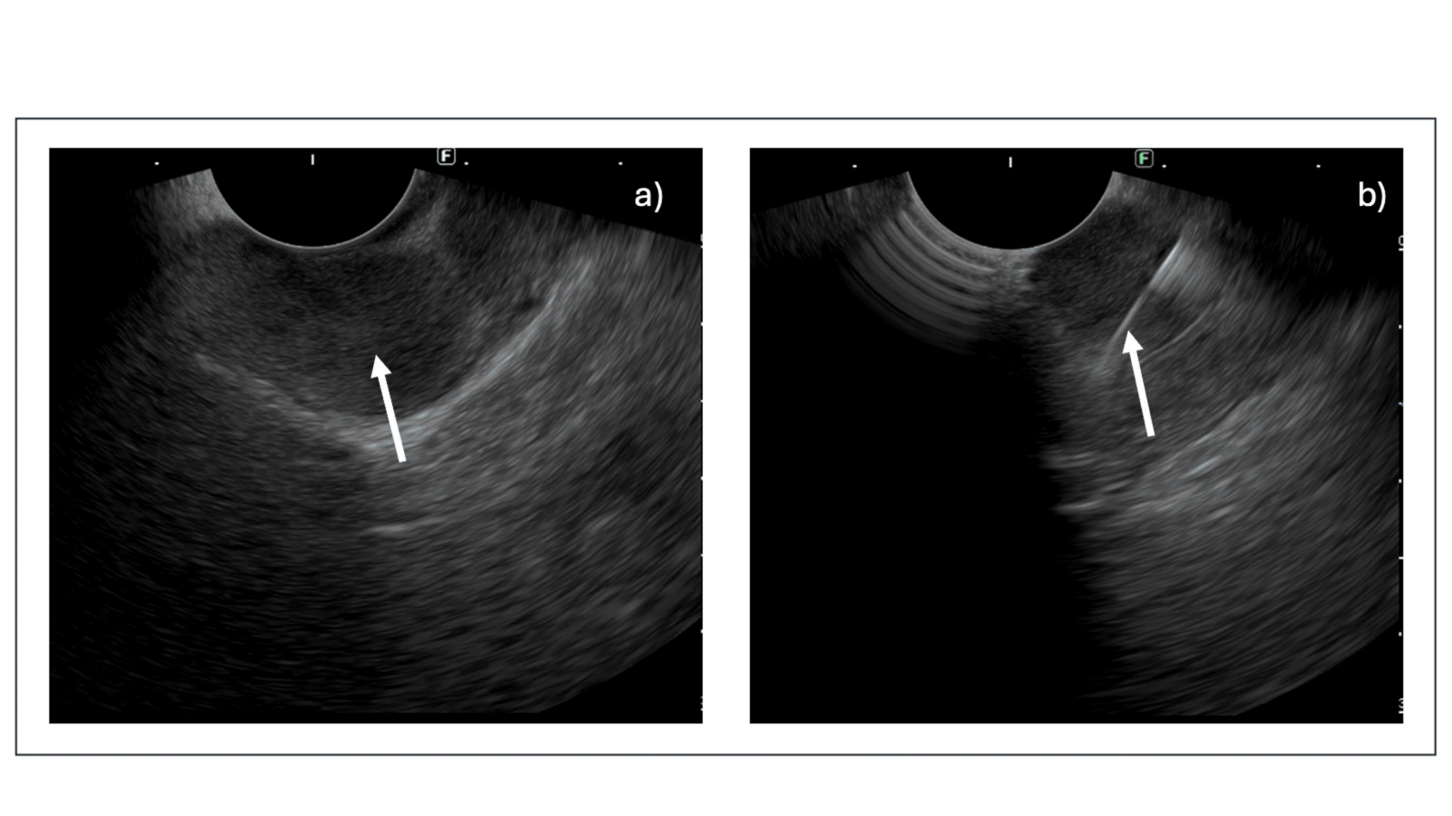
Endoscopic guided ultrasound image showing (a) hypoechoic mass (arrow) in the pancreas and (b) hyperechoic biopsy needle (arrow) within the mass.

Given multiple ulcers in unusual locations and esophagitis being resistant to treatment, serum gastrin levels were sent, and it was found to be 696 pg/mL. Serum chromogranin levels were also elevated (900 pg/ml). Ga-DOTANOC PET CT showed an intense DOTANOC avid hyperattenuating polypoidal lesion of 1.5 x 1.5 cm at the gastroduodenal junction, suggesting somatostatin receptor (SSTR) expression. No tracer avid lesion was identified elsewhere in the body. Endoscopic ultrasound (EUS)-guided biopsy of the lesion was done (Figure 2).

**Figure 3:**
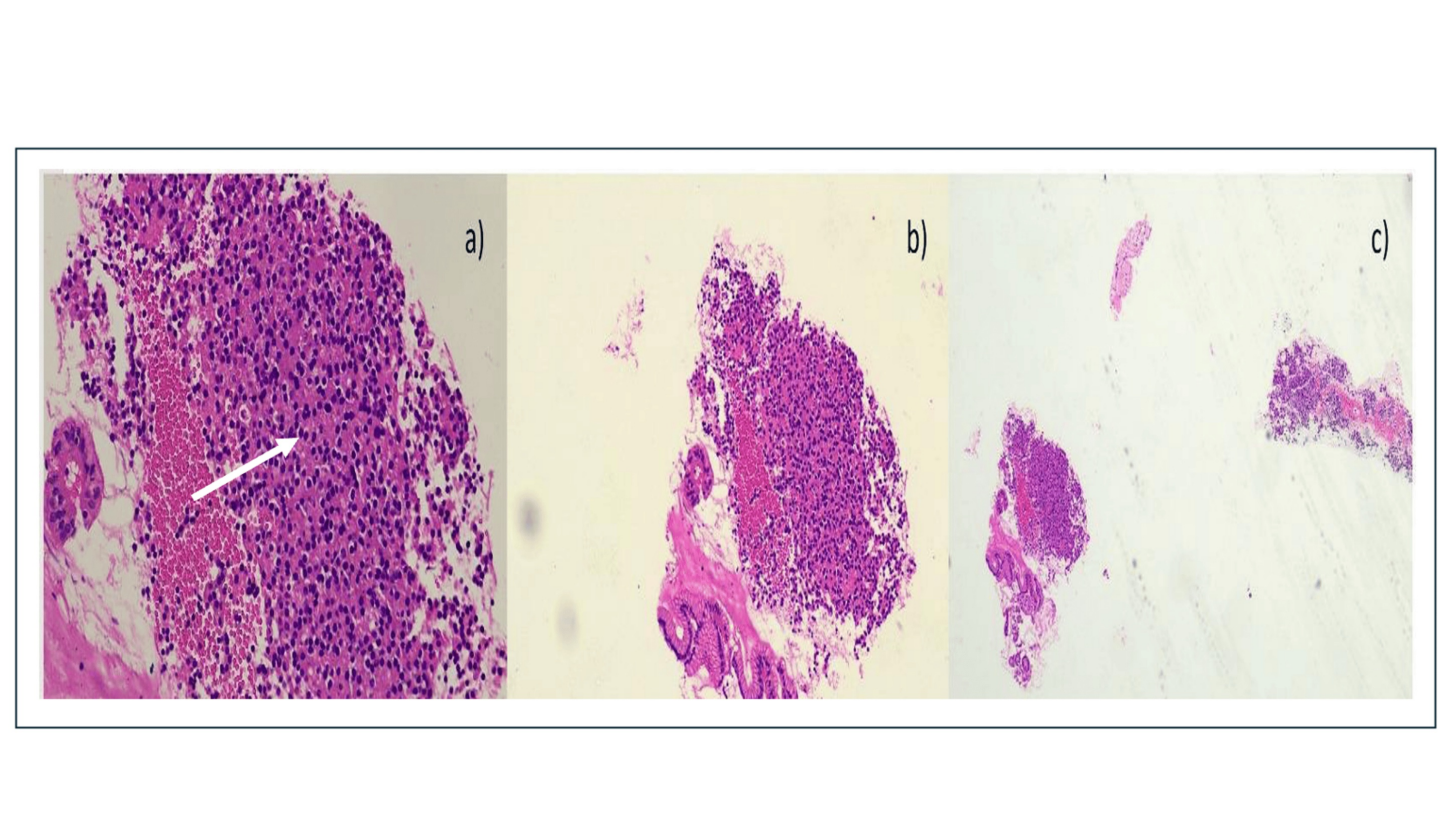
(a-c) Scanty mononuclear round cell clusters with eosinophilic cytoplasm consistent with neoplasm (white arrow).

Histopathology with immunohistochemistry (IHC) studies showed scanty mononuclear round cell clusters with eosinophilic cytoplasm consistent with neoplasm (Figure 3). Cells are diffusely and strongly positive for chromogranin and synaptophysin with a Ki67 index of 3-4%, suggestive of intermediate-grade neuroendocrine tumor. The TNM staging for this patient would be T1 N0 M0, indicating an early-stage, localized neuroendocrine tumor.

The patient was referred for surgical oncology opinion and was started on a four-week course of high-dose pantoprazole 40 mg twice a day and octreotide. During follow-up visits to assess clinical response, the patient endorsed symptom improvement. Follow-up serum gastrin levels have decreased to normal limits, indicating adequate response to the treatment.

## Discussion

ZES is a clinical syndrome caused by excessive gastrin secretion from gastrinoma. Gastrinoma could be associated with MEN1 or be sporadic. MEN1 syndrome is characterized by parathyroid, pancreatic, and pituitary tumors manifesting as hyperparathyroidism, pancreatic NETs, and adenoma, respectively [1]. Patients with gastrinomas associated with MEN1 syndrome are usually diagnosed at younger ages and have better overall survival and progression-free survival rates compared to sporadic cases [9]. In this patient, we found no association between gastrinoma and MEN1. No pituitary or parathyroid involvement was found. In this case, it was initially managed as infective diarrhea with empirical antibiotics and symptomatic management. After two months of persistent symptoms, a UGI endoscopy was done, and it showed oesophagitis and distal gastric hyperemia with prominent mucosal folds. The Rapid Urease test was positive, and she was treated for H pylori gastritis along with symptomatic management. However, stool examination showed no blood, the UGI endoscopy showed no evidence of inflammatory bowel disease, and the duodenal biopsy did not show villous atrophy. Serum Ig-A tissue transglutaminase and Ig-A endomysial antibodies were negative, ruling out coeliac disease. So, the patient was treated with an H. pylori regimen and PPIs. The patient was readmitted to the hospital after a few months in view of the worsening of existing symptoms. The patient has been worked up again in detail. UGI endoscopy was performed again, which showed esophagitis with multiple ulcers over the folds in the duodenum and jejunum, and the colonoscopy was unremarkable. In view of multiple ulcers in unusual locations and oesophagitis being resistant to treatment, gastrinoma was suspected, and serum gastrin levels were sent, which were found to be 696 pg/mL.

Gastrinoma is often suspected when patients have extensive peptic ulcer disease with ulcers in unusual locations [6]. It is mostly observed in the gastrinoma triangle, bounded superiorly by the cystic and common bile ducts, medially by the pancreatic head and body, and inferiorly by the duodenal second and third portions [1,10,11]. Early diagnosis is difficult due to the lack of specific symptoms and the rarity of tumors [1]. Diarrhea may be an additional manifestation in 50% of cases and the only clinical manifestation in 10% of gastrinoma patients [6]. The chief cause of diarrhea is the secretion of large amounts of hydrochloric acid. ZES should be considered in patients with diarrhea and UGI endoscopy showing ulcers in unusual locations [12]. An elevated serum Gastrin concentration can be used as a sensitive indicator of gastrinoma but is not usually specific [12]. In patients with gastrinoma, serum gastrin levels are generally 10 times above normal [1]. After the biochemical diagnosis of gastrinoma, previously, it was not possible to pinpoint the location of the primary tumor. Imaging procedures such as somatostatin receptor scintigraphy scans have improved the accuracy of locating neuroendocrine tumors [10].

Differential diagnoses in patients with chronic diarrhea include vasoactive intestinal peptide-secreting tumors (VIPomas), insulinoma, glucagonoma, somatostatinoma, and carcinoid syndrome [13,14]. Patients with VIPomas classically present with watery diarrhea, hypokalemia, and achlorhydria and have elevated plasma levels of VIP. Occasionally, patients with insulinoma may present with diarrhea secondary to autonomic dysfunction. Diagnosis is based on elevated insulin levels and, on the other most common symptom, hypoglycemia. Glucagonoma has additional clinical manifestations of diarrhea, such as diabetes mellitus and necrolytic migratory erythema. Measuring plasma glucagon levels helps in excluding this tumor from the differential diagnosis. Similarly, somatostatinoma and carcinoid tumors can be excluded through plasma somatostatin and 5-hydroxyindoleacetic acid levels, respectively.

Imaging helps in localizing the tumor, evaluating for disease extent, and for surgical planning. Ultrasound may demonstrate these tumors as round to oval, well-circumscribed hypoechoic lesions with smooth margins. Endoscopic ultrasound has higher sensitivity (94%) in detecting the lesions and additionally helps in obtaining the tissue for histopathological evaluation [11]. On CT, gastrinomas are well-circumscribed lesions with avid enhancement on contrast administration due to their rich capillary network. There may be areas of cystic or calcific degeneration within the larger lesions, which would result in a heterogeneous appearance. Arterial phase images have higher sensitivity in identifying the lesions than portal venous phase images (83%-88 % versus 11%-76 %) [11]. Like the primary tumors, the lymph node and solid organ metastases are highly vascular and more conspicuous on arterial phase images [11]. On MRI, the gastrinomas are more commonly hypointense on T1-weighted and hyperintense on T2-weighted images [11]. The lesions demonstrate intense enhancement on gadolinium administration. Nuclear medicine imaging utilizes the somatostatin receptor expression in gastrinomas. Gallium-68 tagged with DOTA-NOC (Tetraazacyclododecanetetraacetic acid- sodium iodide (NaI) octreotide) or DOTATOC (DOTA-tyrosine-3-octreotide) is used to identify functioning tumors. Sodium iodide octreotide has a higher affinity for SSTR subtypes 2, 3, and 5, while tyrosine-3-octreotide has a higher affinity for SSTR subtype 2 [11]. This nuclear imaging technique has higher sensitivity and specificity of 72-100% and 83-100%, respectively [7].

In our patient, Ga68-DOTANOC PET CT showed a tracer avid polypoidal lesion at the gastroduodenal junction with no evidence of abnormal tracer avid lesions elsewhere in the body, suggesting the tumor localization to the gastroduodenal junction with absent metastasis. EUS-guided biopsy of the lesion was performed to obtain tissue for histopathological examination. The biopsy aimed to characterize the lesion and confirm the presence of a neuroendocrine tumor through microscopic evaluation and immunohistochemical staining. The final diagnosis of gastrinoma depends on pathology and IHC analysis [8]. Histopathology with IHC studies showed scanty mononuclear round cell clusters with eosinophilic cytoplasm consistent with neoplasm. Cells are diffusely and strongly positive for chromogranin and synaptophysin with a Ki67 index of 3-4%, suggestive of intermediate-grade neuroendocrine tumor. The TNM staging for this patient would be T1 N0 M0, indicating an early-stage, localized neuroendocrine tumor with no lymph node or distant metastasis involvement.

The two primary goals of treatment include tumor growth control and symptom relief. Symptom relief can be achieved with proton pump inhibitors. Surgical resection is the potential curative management for gastrinoma [8,10]. Studies show approximately 94% and 34% of a 10-year overall survival and disease-free survival in patients undergoing surgical resection [5]. However, the role of surgical resection is not well-defined in patients with extensive invasive disease or advanced metastasis. In extensive local tumor invasion, surgical resection should still be attempted if there is a chance for at least 80% tumor resection [5]. For hepatic metastasis, liver-directed therapies such as embolization are not frequently applied in the case of gastrinomas, unlike other neuroendocrine tumors [5]. Antiproliferative treatment is recommended in non-surgical candidates with advanced and metastatic disease. Somatostatin analogs such as octreotide and lanreotide reduce the hormone secretion from functioning tumors, thereby providing symptom relief, and are well studied in PROMID and CLARINET randomized control trials [15,16]. Combining chemotherapeutic agents, temozolomide, and capecitabine has shown improved disease control rates in metastatic gastrinomas [17]. Peptide receptor radionuclide therapy is also a promising treatment that uses SSAs labeled with radioisotopes such as 90Yttrium (90Y) or 177 Lutetium (177Lu) [18,19]. Gastrinomas have high response rates to peptide receptor radionuclide therapy (PRRT). However, they also have high recurrence rates [7].

## Conclusions

In summary, gastrinoma should be included in the differentials among the patients presenting with clinical signs of peptic ulcer disease. Further prompt workups with gastrin levels, gastric pH, and UGI endoscopy will aid in these patients' early diagnosis and management. Histopathology helps in making an accurate diagnosis. The two primary goals of gastrinoma treatment include controlling gastric acid hypersecretion and treating the gastrinoma tumor itself. The former can be achieved through proton pump inhibitors, while the latter depends on the tumor staging. Surgical resection is the potential curative treatment for local tumors; however, if the disease is advanced to distant sites, tumor growth control can be achieved through SSAs, chemotherapy, targeted therapies, and PRRT.

## References

[1] Barbosa A, Gomes F, Fonseca L, Maia T, Almeida J (2020). De novo gastrinoma: A case report. GE Port J Gastroenterol.

[2] Jensen RT, Ito T, Feingold KR (2000). Gastrinoma. https://pubmed.ncbi.nlm.nih.gov/25905301/.

[3] Sun QK, Wang W, Zhou HC (2014). Misdiagnosed gastrinoma: A case report. Oncol Lett.

[4] Aamar A, Madhani K, Virk H, Butt Z (2016). Zollinger-Ellison syndrome: A rare case of chronic diarrhea. Gastroenterology Res.

[5] Rossi RE, Elvevi A, Citterio D, Coppa J, Invernizzi P, Mazzaferro V, Massironi S (2021). Gastrinoma and Zollinger Ellison syndrome: A roadmap for the management between new and old therapies. World J Gastroenterol.

[6] Zimmer T, Stölzel U, Bäder M (1995). Brief report: a duodenal gastrinoma in a patient with diarrhea and normal serum gastrin concentrations. N Engl J Med.

[7] Chatzipanagiotou O, Schizas D, Vailas M (2023). All you need to know about gastrinoma today | Gastrinoma and Zollinger-Ellison syndrome: A thorough update. J Neuroendocrinol.

[8] Zimmer V, Schilling MK, Buecker A, Lammert F, Raedle J (2009). Chronic diarrhea responding to proton pump inhibitors: A clinical sign of Zollinger-Ellison syndrome. Am J Med.

[9] Massironi S, Rossi RE, Laffusa A (2023). Sporadic and MEN1-related gastrinoma and Zollinger-Ellison syndrome: Differences in clinical characteristics and survival outcomes. J Endocrinol Invest.

[10] Yang RH, Chu YK (2015). Zollinger-Ellison syndrome: Revelation of the gastrinoma triangle. Radiol Case Rep.

[11] Lewis RB, Lattin GE Jr, Paal E (2010). Pancreatic endocrine tumors: Radiologic-clinicopathologic correlation. Radiographics.

[12] Zhang WD, Liu DR, Wang P, Zhao JG, Wang ZF, Chen LI (2016). Clinical treatment of gastrinoma: A case report and review of the literature. Oncol Lett.

[13] Khan MS, Walter T, Buchanan-Hughes A, Worthington E, Keeber L, Feuilly M, Grande E (2020). Differential diagnosis of diarrhoea in patients with neuroendocrine tumours: A systematic review. World J Gastroenterol.

[14] Pusceddu S, Rossi RE, Torchio M (2020). Differential diagnosis and management of diarrhea in patients with neuroendocrine tumors. J Clin Med.

[15] Caplin ME, Pavel M, Ćwikła JB (2014). Lanreotide in metastatic enteropancreatic neuroendocrine tumors. N Engl J Med.

[16] Rinke A, Müller HH, Schade-Brittinger C (2009). Placebo-controlled, double-blind, prospective, randomized study on the effect of octreotide LAR in the control of tumor growth in patients with metastatic neuroendocrine midgut tumors: A report from the PROMID Study Group. J Clin Oncol.

[17] Bongiovanni A, Liverani C, Foca F (2021). Temozolomide alone or combined with capecitabine for the treatment of metastatic neuroendocrine neoplasia: A "real-world" data analysis. Neuroendocrinology.

[18] Strosberg J, El-Haddad G, Wolin E (2017). Phase 3 trial of (177)Lu-Dotatate for midgut neuroendocrine tumors. N Engl J Med.

[19] Grozinsky-Glasberg S, Barak D, Fraenkel M (2011). Peptide receptor radioligand therapy is an effective treatment for the long-term stabilization of malignant gastrinomas. Cancer.

